# Safety and tolerability of IRL790 in Parkinson’s disease with levodopa-induced dyskinesia—a phase 1b trial

**DOI:** 10.1038/s41531-018-0071-3

**Published:** 2018-12-06

**Authors:** Per Svenningsson, Anders Johansson, Dag Nyholm, Panagiota Tsitsi, Fredrik Hansson, Clas Sonesson, Joakim Tedroff

**Affiliations:** 1 Department of Clinical Neuroscience, Karolinska Institutet, 171 77 Stockholm, Sweden; 2 Department of Neurology, Karolinska University Hospital, 171 76 Stockholm, Sweden; 3 Department of Neuroscience, Neurology, Uppsala University, 751 85 Uppsala, Sweden; 4 Clinical Trial Consultants, Dag Hammarskjöldsväg 13, 752 37 Uppsala, Sweden; 5 Integrative Research Laboratories AB, Arvid Wallgrens backe 20, 413 46 Göteborg, Sweden

## Abstract

IRL790 is a novel compound with psychomotor stabilizing properties primarily targeting the dopamine D3 receptor. IRL790 is developed as an experimental treatment for levodopa-induced dyskinesia (LID), impulse control disorder, and psychosis in Parkinson’s disease (PD). The primary objective was to investigate the safety and tolerability of IRL790 in PD patients with LID in a randomized controlled trial. PD patients with peak-dose dyskinesia were randomized to placebo or IRL790 treatment (1:3 ratio) for 4 weeks. Study drug was given as an adjunct treatment to the patients’ regular stable antiparkinsonian medication. Dosing was individually titrated for 14 days, whereafter dosing was kept stable for an additional 14 days. Fifteen patients were randomized to treatment and 13 patients completed the 4-week treatment. Adverse events were mostly reported during the titration phase of the trial. They were mainly central nervous system related and could be mitigated by dose adjustments. There were no serious adverse events. There were no clinically significant changes in vital signs, electrocardiogram, and laboratory parameters due to the treatment. The average dose in the stable dose phase was 18 mg daily, yielding a 2-h post-dose plasma concentration of average 229 nM on day 28. Assessments for motor function showed a numeric reduction in dyskinesia. It is concluded that IRL790 can be safely administered to patients with advanced PD. The results will be of guidance for the design of phase 2 studies.

## Clinical trial: New drug counters dyskinesia

A compound that blocks the dopamine D3 receptor alleviates the abnormal involuntary movements caused by long-term treatment with levodopa. Nearly 50% of Parkinson’s disease (PD) patients treated with levodopa for more than 5 years develop levodopa-induced dyskinesia. Previous studies have shown an increase in the levels of D3 receptor in the dorsal striatum of patients experiencing this motor complication. Svenningsson at the Karolinska Institutet, Sweden, and colleagues examined the safety and tolerability of IRL790 in patients with advanced PD and levodopa-induced dyskinesia. After four weeks, no serious adverse events were observed and dyskinesia was reduced in patients receiving IRL790 along with their regular antiparkinsonian medication. Further trials will help determine the optimal dose of IRL790 and whether it could be used to treat other adverse effects associated with long-term levodopa use such as impulse control disorder.

## Introduction

Motor complications, including levodopa-induced dyskinesias (LID), affect nearly half of the patients with Parkinson disease (PD) treated with levodopa in the first 5 years of treatment.^1,2^ Several mechanisms underlying the development of motor complications, such as LID, have been proposed.^3,4^ It has repeatedly been shown that chronic treatment with levodopa induces an increase in dopamine D3 receptor (D3R) expression in the dorsal striatum in rats with 6-hydroxydopamine (6-OHDA) lesions^5,6^ and non-human primates rendered parkinsonian with 1-methyl-4-phenyl-1,2,3,6-tetrahydropyridine (MPTP).^7^ This increase correlates with LID.^7,8^ Moreover, studies with D3R partial agonists or antagonists or D3R knockout mice also suggest that D3R deletion significantly attenuates LID.^7,9^ Interestingly, nigrostriatal dopaminergic deficiency does not seem to be a prerequisite for LID, since levodopa treatment could induce abnormal involuntary movements in intact rodents overexpressing D3Rs in the dorsal striatum.^10^ In patients with PD, positron emission tomography (PET) using the D3R preferring radioligand [^11^C]PHNO, has demonstrated increased binding in the dorsal striatum in levodopa-treated patients, and a further elevation of tracer binding in the globus pallidus in patients with LID.^11^

IRL790 belongs to a new class of central nervous system (CNS) active agents called psychomotor stabilizers. Such compounds modify psychomotor activity depending on the initial level of activity. In vitro, IRL790 acts as an antagonist of brain neuroreceptors belonging to the dopamine D2-type (D2 and D3) receptors with a strong preference for the D3R (Ki = 90 nM) versus D2R (Ki = 850 nM). In 6-OHDA lesioned rats rendered dyskinetic by prolonged levodopa treatment, IRL790 dose-dependently suppresses abnormal involuntary movements without compromising the ability for forward locomotion.^12^ In preclinical models, IRL790 also displays antipsychotic properties.^12^ Taken together, the preclinical pharmacology of IRL790 indicates a novel profile with a potential to alleviate adverse effects associated with long-term levodopa treatment in PD.

IRL790 has previously undergone safety and tolerability testing in healthy male volunteers. The present study was undertaken to study the safety and tolerability of adjunct IRL790 treatment in the intended patient population.

## Results

**Fig. 1:**
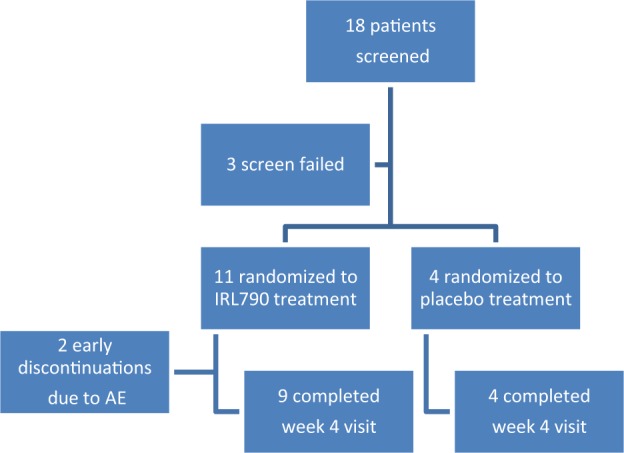
Overview of trial profile

Between November 2016 and March 2017, 18 patients were screened and 15 patients were randomized (Fig. 1). Demographics and baseline treatment of the intention-to-treat (ITT) population are shown in Table 1.

**Table 1:** Baseline demographics and treatment for the intention-to-treat (ITT) population

	IRL790, *n* = 11	Placebo, *n* = 4
Age, years	71.2 (6.4)	65.5 (10.7)
Sex, male/female	6/5	3/1
Years since PD diagnosis	13.7 (5)	14.2 (9)
*Concomitant antiparkinsonian medication uses at baseline*		
Oral levodopa	9	3
Levodopa intestinal gel infusion	1	1
Apomorphine infusion + oral levodopa	1	–
COMT inhibitor	4	1
MAO-B inhibitor	4	1
Dopamine agonist	8	3
Amantadine	4	1
Anticholinergic	–	1

Following dose titration, the average dose titrated for placebo patients during the last 2 weeks of treatment was 42 mg (20–80 mg) daily. For IRL790-treated patients, the average dose was 18 mg (10–30 mg) daily. Three patients ended up taking 10 mg once daily. Since the protocol stated the lowest dose to 10 mg b.i.d. and the highest to 40 mg b.i.d., these doses were in violation with the protocol. However, it was agreed that these dose adjustments constituted minor protocol deviations and were thus allowed. Eight patients (72.8%) treated with IRL790 and one (25.0%) of the placebo patients had the dose reduced due to an adverse event (AE) at any time during the first 14 days of treatment. Listings of dose decisions and related AEs are summarized in Supplementary Table 1.

**Fig. 2:**
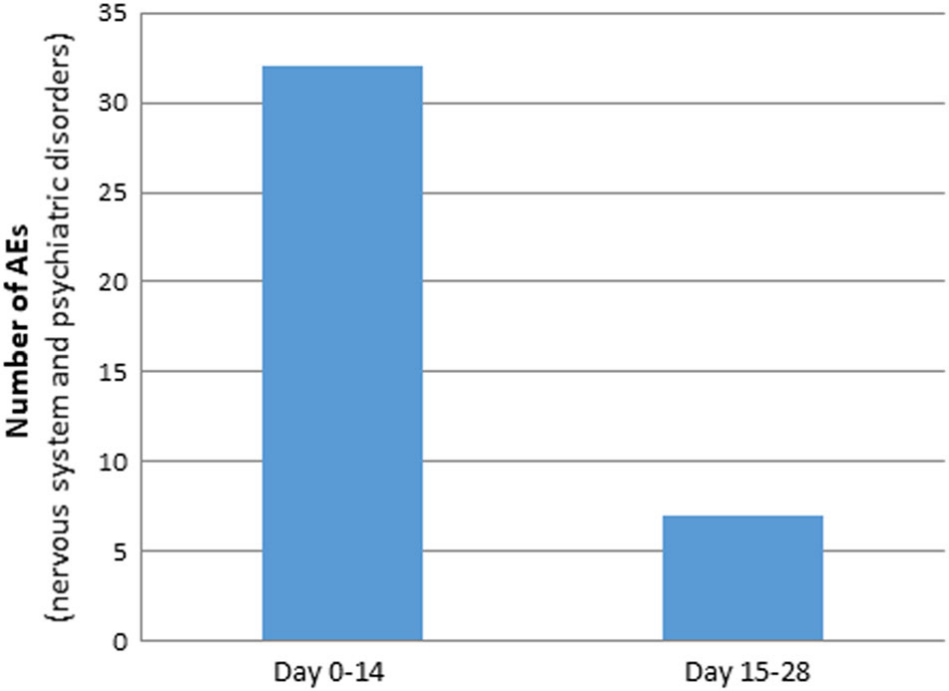
Number of adverse events (AEs) belonging to the system organ classes nervous system and psychiatric disorders reported during the titration phase (day 1–14), and following stable dosing (day 15–28)

A total of 62 AEs was reported by 14 patients (93.3%). The total number of AEs was 41 when counting an event term only once per patient (i.e., patient unique AEs). Most AEs were reported during the titration phase (first 14 days of treatment), 48 AEs were reported between day 1 and 14 of treatment (titration phase), and 14 AEs between day 14 and follow-up. For system organ class (SOCs) belonging to nervous system disorders and psychiatric disorders, patients treated with IRL790 reported 33 AEs between day 1 and 14 of treatment and 8 AEs between day 14 and follow-up (Fig. 2).

All patients treated with placebo experienced at least one AE, as compared with 90.1% of the patients treated with IRL790. Approximately 80% of the patients in both groups experienced at least one AE assessed as related to the investigational medicinal product (IMP). No seroius adverse events (SAEs) occurred in the study. Most of AEs (73%) were of grade 1 (mild) intensity. Most AEs (52%) reported were considered as not related to study treatment. AEs considered related to study drug administration were mostly CNS related, mostly mild, and transient and could be mitigated by dose adjustments. Two patients (18.2%) in the IRL790 group were withdrawn due to AEs assessed as possibly related to study treatment. One patient withdrew due to dyspnea and dizziness and the other patient due to edema and redness of the feet. On retrospective review, both patients had a previous history of such symptoms. A summary of AEs is found in Table 2.

**Table 2:** Adverse events overview

	IRL790	Placebo
*Number (%) of participants with any*		
AE	10 (90%)	4 (100%)
Serious AE	0	0
Study drug‐related serious AEs	0	0
*Number (%) of participants who permanently discontinued treatment because of any*		
AE	2 (18%)	0
*Most common AEs (number of patients with an AE reported by more than one patient, by treatment groups):*		
Worsening of parkinsonism	5	1
Headache	1	1
Asthenia	2	–
Fatigue	1	1
Dissociation	3	–

Except from a spontaneous fracture in the left shoulder (day 28) in one patient (considered non-related to study medication), no abnormal physical examination findings were assessed as clinically significant at any of the time points assessed. There were no clinically relevant mean changes over time with regard to any of the vital signs parameters. Mild hypertension assessed as possibly related to study treatment was reported on days 11 and 13 of study treatment for one patient treated with IRL790. No other individual abnormal values were assessed as clinically significant. There were no clinically relevant mean changes over time or individual clinically significant abnormal values with regard to any of the electrocardiogram (ECG) parameters from the start of study treatment.

A slight increase in prolactin levels was observed after treatment with IRL790. The mean relative change from baseline (screening) to day 28 of treatment was 71.5 (71.8)% (median 58.5) in the IRL790 group, as compared with 28.4 (59.0)% (median 7.1) in placebo-treated patients. There were no individual abnormal prolactin values assessed as clinically significant. Moderate thrombocytopenia assessed as possibly related to study treatment was reported at follow-up in one patient treated with IRL790. No other abnormal values were assessed as clinically significant during the study. There were no clinically relevant changes in mean values or individual changes over time for any of the other safety laboratory parameters analyzed.

### Efficacy results

Efficacy assessments were made at baseline and following stable dose treatment. In IRL790-treated patients, a consistent numeric reduction in dyskinesia assessments was seen. On the Unified Dyskinesia Rating Scale (UDysRS), a median reduction of 11,5 points vs. placebo and a mean reduction of 8,2 points vs. placebo was observed for the IRL790-treated group (ITT population) during the 4-week study. Among patients treated with IRL790, 55.5% were assessed as having an improved global clinical condition, as compared with baseline (much improved/minimally improved). There were no changes in symptoms relating to parkinsonism, either in the Unified Parkinson’s Disease Rating Scale (UPDRS) or Parkinson’s Kinetigraph^TM^ assessments. Descriptive results from efficacy assessments are shown in Table 3.

**Table 3:** Descriptive statistics of efficacy assessments; intention-to-treat (ITT) population

	IRL790				Placebo			
	Median		Mean (SD)		Median		Mean (SD)	
	Baseline	Week 4	Baseline	Week 4	Baseline	Week 4	Baseline	Week 4
UDysRS	33	23*	33 (12)	22.6 (9)*	35	36.5	39.5 (19)	37.25 (14)
UPDRS 4 q 32–35	4	2	4.4 (2)	2.1 (1)	4.5	3.5	4.5 (1)	3.5 (2)
PKG, dyskinesia	6.8	3.9	5.6 (3)	3.9 (3)	6.7	4.7	11.8 (12)	6.3 (6)
PKG, bradykinesia	25.5	24.6	24.5 (5)	26.2 (6)	17.6	20.1	17.9 (4)	19.9 (2)
UPDRS part 1	3	2	2.8 (2)	2.8 (2)	3	2	3.2 (1)	3.0 (2)
UPDRS part 2	13	10	12 (5)	10.4 (5)	13	12	14.2 (8)	13.8 (8)
UPDRS part 3	14	14	18.2 (10)	15.7 (5)	19	16	19.8 (7)	15 (2)
UPDRS part 4	9	6	7.8 (2)	6.3 (2)	8.5	8	8.7 (2)	7.5 (3)

*P < 0.01; Wilcoxon signed rank sum test

### Pharmacokinetic results

Pharmacokinetic (PK) analysis showed peak plasma concentrations within the expected range (expectedness based on previous studies in healthy male volunteers). Based on mean plasma concentrations of IRL790 and its metabolites, a steady state seemed to have been reached after 14 days of treatment. Table 4 shows summary statistics of plasma concentrations of IRL790 at pre-dose and 2 h after drug administration at baseline, day 14 and day 28 of treatment.

**Table 4:** Summary statistics over time for plasma IRL790 concentrations at day 1, 14, and 28 of treatment

		Concentration IRL790 (nM)								
		*n*	Mean	SD	Min	Q1	Median	Q3	Max	Missing
Day 1	120 min post dose	11	155.91	26.63	123.00	136.00	145.00	186.00	198.00	0
Day 14	Pre-dose	8	81.34	59.46	21.60	27.80	76.75	113.50	193.00	1
	120 min post dose	9	244.00	117.17	145.00	159.00	218.00	290.00	515.00	0
Day 28	Pre-dose	9	58.20	32.66	20.80	34.00	53.40	64.90	124.00	0
	120 min post dose	9	229.44	90.64	142.00	163.00	198.00	250.00	431.00	0

## Discussion

Motor and mental complications associated with long-term antiparkinsonian treatment remain a significant problem in the management of PD. IRL790 belongs to a novel class of compounds with psychomotor stabilizing properties demonstrated in experimental animals. IRL790 decreases psychomotor activity in states of abnormally high activity (such as after amphetamine challenge, glutamate blockade by MK801, or in LID), but also stimulates activity in states of low psychomotor tone such as following environmental habituation.^12^ It is widely accepted that pulsatile stimulation of dopamine receptors in the denervated striatum alters neuronal firing patterns and motor output from the basal ganglia ultimately leading to dyskinesia. It is suggested that this process involves overexpression of D3Rs in the output projections of the basal ganglia, as shown in experimental animals,^5,8^ as well as in patients with PD.^11^ IRL790 exerts its efficacy primarily via interaction with D3R (antagonist, Ki about 90 nM) and as such may be useful to treat not only LID but also several other complications of therapy in PD, where D3R dysfunction is implicated.^13–16^

A previous phase 1 study in healthy male volunteers demonstrated that IRL790 was well-tolerated in single doses up to 120 mg and multiple daily doses up to 80 mg (manuscript in preparation). The present study shows that patients with advanced PD tolerate lower doses of IRL790, as compared with younger healthy male volunteers. The most frequent AEs were CNS related and expected to be due to the pharmacology of IRL790. Five patients on IRL790 and one on placebo experienced a slight increase in parkinsonism during titration (days 1–14). It was transient and could be mitigated by dose adjustments, but needs attention in future phase 2 studies. Assessments for dyskinesia obtained by the UDysRS, questions 32–35 of the UPDRS part 4, and data obtained from the PKG device all indicated that IRL790 treatment reduced dyskinesia as compared with baseline assessments. Assessments for motor function and bradykinesia following individual dose titration indicated no general worsening of parkinsonism with treatment following dose titration. Adjuvant treatment with IRL790 in PD seems to be safe; no cardiovascular adverse effects were reported or detected. Laboratory assessments were essentially unaltered, except for a slight increase in prolactin, which is an expected pharmacodynamic effect of IRL790. PK analysis showed peak plasma concentrations within the expected range, and no unexpected accumulation occurred over the 4 weeks of treatment. Peak plasma concentrations following dose titration were in a range corresponding to 2–3 times of the Ki for IRL790 binding to D3Rs obtained in vitro. Further clinical studies with PET would need to be done to establish D3R occupancy at therapeutic plasma concentrations in patients with PD. Four IRL790-treated and one placebo-treated patient were on amantadine therapy. Since IRL790 and amantadine counteract LIDs via different mechanisms, they may act synergistically. This needs to be addressed in larger clinical trials and in preclinical studies. In conclusion, IRL790 can be safely administered to patients with advanced PD. However, the optimal dose of IRL790 in PD patients appears to be lower than anticipated from dose titration studies in healthy volunteers. The results from this study will be of guidance for the design of further efficacy studies.

## Methods

### Study design and participants

A phase 1b, randomized, double‐blind, placebo‐controlled study (Integrative Research Laboratories AB) was conducted at three university hospital outpatient clinics in Sweden. The study was conducted in accordance with the Declaration of Helsinki and Good Clinical Practice Guidelines. Prior to initiating the study, the protocol underwent ethical vetting and all participating sites received approval from the central ethical review board in Stockholm, Sweden. Written informed consent was obtained from all study participants before any study‐related procedures were performed. The ClinicalTrials.gov Identifier is NCT03531060.

Key inclusion criteria included age between 50 and 85 years, diagnosis of PD based on the United Kingdom Parkinson’s Disease Society Brain Bank Clinical Diagnostic Criteria, and showing a clear peak-dose dyskinetic response to regular levodopa medication. All antiparkinsonian medications, including levodopa preparations, were to be unchanged for at least 30 days prior to screening and during study participation.

Key exclusion criteria included history of any clinically significant disease or disorder which, in the opinion of the investigator, could either put the patient at risk because of participation in the study, or influence the results or the patient’s ability to participate in the study; history of or present clinically significant psychiatric diagnosis, at discretion of the investigator; history of seizures, including febrile seizure in childhood; history or presence of hepatic or renal disease or other condition known to interfere with the absorption, distribution, metabolism, or excretion of drugs; previous surgery for PD; a Hoehn and Yahr score of 5 when “off” prolonged QTcF (>450 ms), cardiac arrhythmias, or any clinically significant abnormalities in the resting ECG at the time of screening, as judged by the investigator; history of severe allergy/hypersensitivity or ongoing allergy/hypersensitivity, as judged by the investigator; or history of hypersensitivity to drugs with a similar chemical structure or class to IRL790.

### Randomization and blinding

The study patients were randomized to treatment with either IRL790 or placebo (3:1). The randomization list was generated by the Clinical Research Organization (CRO). The original randomization list was kept in a sealed envelope at the CRO and a copy at the hospital pharmacy. Sealed treatment code envelopes were kept by each site. The individual doses were dispensed to the patients at the clinics as per the randomization list. Data analyzers were blinded until after the database was locked. No code envelope was broken during the study.

### Procedures

Consenting patients were screened for eligibility as per study-specific inclusion/exclusion criteria within 8–28 days before the start of treatment (Visit 1; Screening Visit). At Visit 2 (day 7) a kinetigraph device (PKG, Global Kinetics Corporation, Melbourne, Victoria, Australia) was attached to the right or left wrist (the parkinsonian dominant side) and baseline patient movement data were recorded during a run-in period of seven consecutive days. Following baseline assessments at Visit 3 (day 1), patients were randomized to receive IRL790 or placebo (3:1). The treatment allocation was double-blinded, i.e., it was not disclosed to the patients, the site staff, or the sponsor. Study medication was dispensed, and the first capsule was administered at the clinic. During the treatment period, clinic visits were performed on days 4, 7, 10, 14, and 28 (end of treatment) and a follow-up phone call was performed on day 21.

All patients were prescribed a dose of 10 mg b.i.d. as the start dose, and individual dose adjustments could be made up to a maximum of 40 mg b.i.d. until day 14 following predefined criteria, and the treating physician’s evaluation of tolerability.

On days 14 and 28, the morning dose was administered at the clinic. ECG was assessed 2 h after dose administration. Blood samples for PK analysis were collected pre-dose and 2 h post dose. During the telephone follow-up call on day 21, the patient was asked to attach the PKG to the same wrist as during run-in and to wear the device until the end of treatment visit on day 28. Ratings for UDysRS and UPDRS were performed at the baseline and day 28 of treatment. The UDysRS was performed 1–2 h after the patient had received 150% of the regular levodopa dose and agreed to be ON. The levodopa dose and post-dose time for assessments were the same at baseline and day 28 for a given individual. On day 28, a Clinician’s Global Impression of Change (CGI‐C) was also administered. Standard safety assessments were performed at weeks day 14 and 28. A follow-up visit was performed 7–10 days after the last dose.

### Outcomes

The primary outcomes of the trial were safety and tolerability observations, such as frequency, seriousness and intensity of AEs, physical examination, ECG recordings, vital signs, and safety laboratory measurements. Secondary outcome measurements related to efficacy were UDysRS, UPDRS parts 1–4 from baseline to day 28, CGI-C at day 28, and the median bradykinesia score and median dyskinesia score as recorded with the PKG from run-in to the last week of treatment.^17^

### Statistical analysis

The ITT population consisted of all patients who were randomized and received at least one dose of study drug. All protocol violations were presented and discussed at the clean file meeting. AEs and SAEs were recorded from the start of study drug administration. Medical events occurring between screening and the first treatment were reported separately as baseline events. All AE data were fully listed by investigator terms and Medical Dictionary for Regulatory Activities (MedDRA) preferred term. AE data were summarized by SOC and the preferred term. PK and efficacy data were presented with summary statistics. No formal sample size calculation was performed for this study and thereby no hypothesis testing. The sample size was considered sufficient to provide adequate information for the study objectives. A post hoc analysis of obtained data before and after IRL790 was made with Wilcoxon SIGNED rank sum test.

## Electronic supplementary material

Supplementary Table 1

## Data Availability

The clinical protocol and datasets generated during and/or analyzed during the current study are available from the corresponding author on reasonable request.

## Electronic supplementary material

**Supplementary information** accompanies the paper on the *npj Parkinson’s Disease* website (10.1038/s41531-018-0071-3).
