# Supplementary material for: Safety and tolerability of IRL790 in Parkinson’s disease with levodopa-induced dyskinesia—a phase 1b trial
Source: NPJ Parkinsons Dis. 2018 Dec 6;4:35. doi: 10.1038/s41531-018-0071-3 (PMC6283839; doi:10.1038/s41531-018-0071-3)
Supplement: Supplementary file 1 [file 41531_2018_71_MOESM1_ESM.docx]

Patient No	Dose	Visit 4	Action	Dose	Visit 5	Action	Dose	Visit 6	Action	Dose	Visit 7	Action	Dose	Visit 9	Visit 10
	mg	Day 4		mg	Day 7		mg	Day 10		mg	Day 14		mg	Day 28	Follow-up
1001	20	Headache	Dose reduced	10	Feeling disconnected	Dose maintained	10	Headache	Dose maintained	10		Dose maintained	10	Headache	
		Worsening of Parkinsonism			Worsening of Parkinsonism			Feeling disconnected						Nightmare	
		Feeling disconnected						Feeling weak							
1002	20	Worsening of Parkinsonism	Dose maintained	10	Loss of energy	Dose reduced	10		Dose maintained	10	High blood pressure	Dose maintained	10	High blood pressure	
					Inner tremor						Worsening of Parkinsonism			Back pain	
														Fall	
1003	20		Dose increased	40	Worsening of Parkinsonism	Dose reduced	20		Dose increased	30	Tinnitus	Dose reduced	20	Worsening of Parkinsonism	
											Worsening of Parkinsonism			1.5 h after IMP intake	
											Feeling disconnected				
1005	20	Feeling loss of bodily control	Dose reduced	10	Worsening of Parkinsonism	Dose maintained	10		Dose maintained	10		Dose maintained	10		
					Vertigo										
					Feeling disconnected										
					Fatigue										
1008	20	Restlessness	Dose maintained	20		Dose increased	40		Dose reduced	20	Confusion	Dose maintained	20	Cognitive deficit	
											Worsening of dyskinesia			Balance impairment	
											Dizziness				
1009	20	Dyspnea	Dose reduced	10		Dose maintained	10	Discontinued							
		Sense of leg weakness													
1012	20		Dose maintained	20		Dose maintained	20		Dose mintained	20		Dose maintained	20	Spontaneous fracture	
														left shoulder	
1013	20		Dose increased	30	Dizziness othostatism	Dose reduced	20	Oedema Red feet (Raynaud)	Dose reduced	Discontinued					Trombocytopenia
1015	20		Dose maintained	20	Insomnia	Dose increased	30		Dose increased	40	Increased rigidity	Dose reduced	30		
											Seasonal flu				
1016	20		Dose increased	40	Worsening of Parkinsonism	Dose reduced	20		Dose maintained	20		Dose maintained	20		
1010	20		Dose maintained	20		Dose maintained	20		Dose maintained	20		Dose maintained	20		
